# Supplementary material for: Spinal cord stimulation modulates post-synaptic inhibition to improve arm neuromotor control after chronic stroke
Source: Cell Rep Med. 2026 Jul 17;7(8):102925. doi: 10.1016/j.xcrm.2026.102925 (PMC13522750; doi:10.1016/j.xcrm.2026.102925)
Supplement: Supplementary file 1 [file mmc1.pdf]

Cell Reports Medicine, Volume *7*

Supplemental information

Spinal cord stimulation modulates post-synaptic inhibition to improve arm neuromotor control after chronic stroke

Luigi Borda, Nikhil Verma, Erynn Sorensen, Erick Carranza, Roberto de Freitas, Scott Ensel, Amy Boos, Peter C. Gerszten, Daryl P. Fields, George F. Wittenberg, Lee E. Fisher, Elvira Pirondini, Marco Capogrosso, and Douglas J. Weber

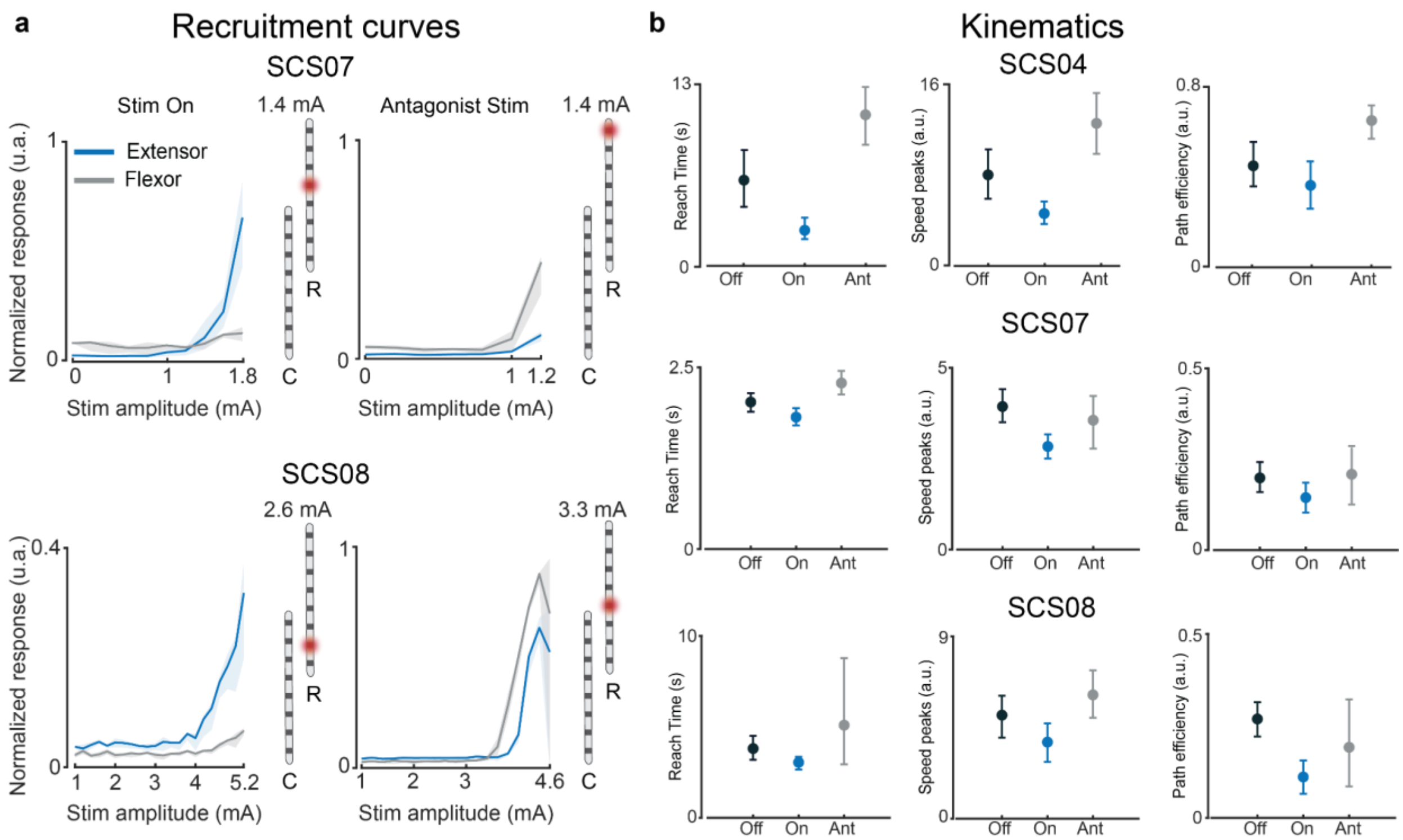

Figure S1 | Planar reaching kinematics and muscle activity, related to Figure 2: a, Top: recruitment curves for contact 4R (left) and contact 1R (right) and normalized evoked responses for triceps brachii (blue, elbow extensor) and biceps brachii (gray, elbow flexor). Bottom: recruitment curves for contact 7R (left) and contact 5R (right) and normalized evoked responses for triceps brachii (blue, elbow extensor) and brachioradialis (gray, elbow flexor). **b**,Quantification of the mean values of the kinematics metrics used to assess the effect of SCS (On) and Sham stimulation with respect to stimulation Off for the three participants.

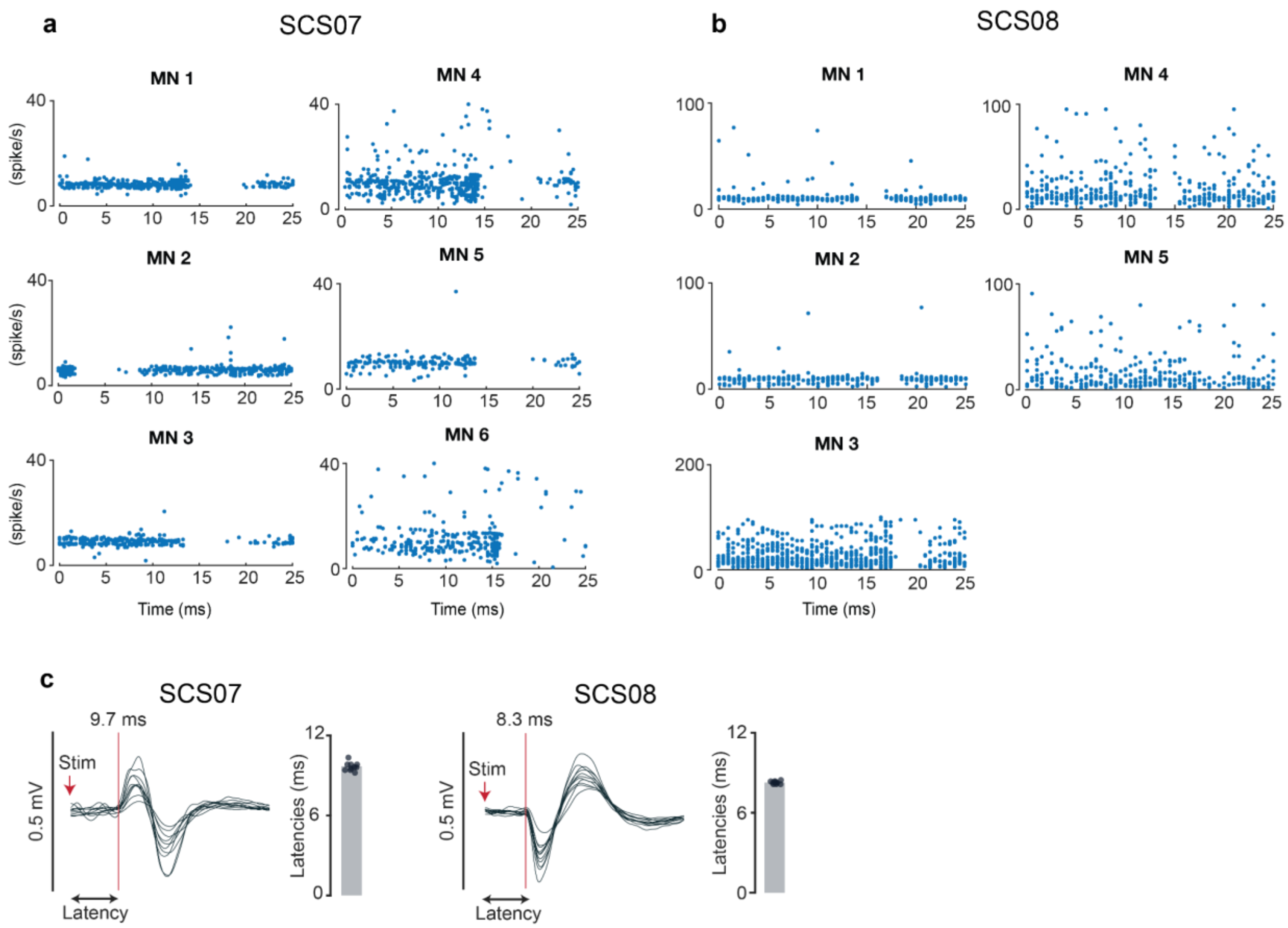

Figure S2 | Peristimulus frequencygram (PSF) and action potential latencies, related to Figure 5: a,**b** Peristimulus frequencygram of all the detected motoneurons (MNs) for SCS07 and SCS08, respectively. Circles on plots indicate the instantaneous firing rate of the MNs at the reported delay from the SCS pulses. The interval considered spans 0 ms to 25 ms post-stimulus, which is the stimulation time period for the 40 Hz tonic SCS used in this experiment. **c**, Latencies of the PRM-reflexes evoked in the biceps brachii as an estimate of the action potential propagation delay for SCS07 and SCS08. Bar plots show the mean value of the action potential delay with respect to the SCS pulses. Circles on plots indicate the time delay between the stimulation pulse and the recorded action potential for n = 10 evoked responses. Evoked responses were recorded at rest while delivering supra-threshold SCS targeting Ia sensory afferents innervating Biceps Brachii.
